# Electronic Excitation-Induced Modification in Electronic Structure and Magnetism for Pulsed Laser Deposited Barium Strontium Titanate Thin Films with Changing Fe Impurity

**DOI:** 10.3390/ma18112534

**Published:** 2025-05-28

**Authors:** Arkaprava Das, Carla Bittencourt

**Affiliations:** Chimie des Interaction Plasma Surface, Research Institute for Materials Science and Engineering, University of Mons, 7000 Mons, Belgium; arkaprava.das@umons.ac.be

**Keywords:** ion irradiation, X-ray photoelectron spectroscopy, electronic energy loss, bound magnetic polaron

## Abstract

This study presents a comprehensive analysis of the modifications in electronic structure and magnetism resulting from electronic excitation in pulsed laser-deposited Ba_0.7_Sr_0.3_Fe_x_Ti_(1−x)_O_3_ thin films, specifically for compositions with x = 0, 0.1, and 0.2. To investigate the effects of electronic energy loss (*S_e_*) within the lattice, we performed 120 MeV Ag ion irradiation at varying fluences (1 × 10^12^ ions/cm^2^ and 5 × 10^12^ ions/cm^2^) and compared the results with those of the pristine sample. The *S_e_* induces lattice damage by generating ion tracks along its trajectory, which subsequently leads to a reduction in peak intensity observed in X-ray diffraction patterns. Atomic force microscopy micrographs indicate that irradiation resulted in a decrease in average grain height, accompanied by a more homogeneous grain distribution. X-ray photoelectron spectroscopy reveals a significant increase in oxygen vacancy (V_O_) concentration as ion fluence increases. Ferromagnetism exhibits progressive deterioration with rising irradiation fluence. Due to the high *S_e_* and multiple ion impact processes, cation interstitial defects are highly likely, which may overshadow the influence of V_O_ in inducing ferromagnetism, thereby contributing to an overall decline in magnetic properties. Furthermore, the elevated *S_e_* potentially disrupts bound magnetic polarons, leading to a degradation of long-range ferromagnetism. Collectively, this investigation elucidates the electronic excitation-induced modulation of ferromagnetism, employing Fe impurity incorporation and irradiation techniques for precise defect engineering.

## 1. Introduction

The concurrent presence of ferromagnetism and electrical polarization in materials at room temperature (RT) and above is rarely reported [1]. Researchers have employed various methodologies, including doping, nano-structuring, and ion irradiation, to tailor ferroic ordering and develop devices that utilize magneto and electrostriction to manipulate polarization or transition temperatures. In the context of advancing data storage technologies, chip-based memory and logic devices are of paramount importance. Consequently, significant attention has been directed toward magnetic thin films, which offer the potential advantage of coexisting ferroelectricity. Over the past few decades, significant progress has been achieved in synthesizing such materials in thin films using the pulsed-laser deposition (PLD) method [2]. Materials such as PbTiO_3_ and PbZrTiO_3_ have been reported as lead-based ferroelectric materials exhibiting high dielectric constants [3]. However, due to the volatile and hazardous nature of lead oxide, researchers are actively seeking alternatives [4]. To address these limitations, barium strontium titanate (BST) has emerged as a promising candidate, exhibiting paraelectric behavior at RT. BST combines the high permittivity from BaTiO_3_ with the structural stability of SrTiO_3,_ rendering it a potential candidate for device applications [5]. In this context, iron (Fe) cationic impurities are typically introduced into the BST solid solution, substituting titanium (Ti) atoms, and are expected to induce ferromagnetism while maintaining the integrity of the perovskite phase. Prior studies have shown that BaFe_x_Ti_1−x_O_3_ bulk samples for 0.06 ≤ x ≤ 0.84 stabilize in hexagonal structures [6]. Guo et al. reported the coexistence of ferroelectricity and ferromagnetism in Fe-doped Ba_0.5_Sr_0.5_TiO_3_ bulk samples [7]. Kaur et al. investigated the electronic structure of Fe-doped Ba_0.7_Sr_0.3_TiO_3_ bulk samples with doping levels of 10%, 20%, and 30%, observing weak ferro- and ferri-magnetic ordering alongside tetragonality [4]. Pulsed laser-deposited BaFe_x_Ti_1−x_O thin films (0.5 ≤ x ≤ 0.75) have demonstrated a pseudo-cubic perovskite structure [1,8]. Consequently, the properties of thin films differ substantially from their bulk counterparts. To compare the electronic and physical properties of bulk and thin film systems, we have synthesized PLD thin films with the same stoichiometry reported by Kaur et al. [4]. In addition to Fe doping, significant enhancements in magnetic properties were achieved through ion beam irradiation techniques [9]. Such irradiation treatments have been applied to Fe–Pt, Ni–Fe, and other alloy thin film systems, which hold considerable significance in the domain of magnetic information storage [9]. Thus, controlled defect engineering with irradiation treatment has also been implemented in the study to modulate the magnetic properties for PLD Ba_0.7_Sr_0.3_Fe_x_Ti_1−x_O_3_ thin films.

When a swift heavy ion (SHI) irradiates a thin film, it traverses the matrix, with the depth penetration dependent on the energy and mass of the ion. During its passage through the material, the ion can ionize atoms through inelastic collisions or displace them via elastic collisions. In the low energy regime of several MeV, the elastic collision, i.e., nuclear energy loss (*S_n_*), dominates, whereas in the high energy regime of a few hundred MeV, the inelastic collision, i.e., electronic energy loss (*S_e_*), becomes predominant. The *S_e_* can generate ion tracks if the temperature within the material exceeds the melting temperature, with subsequent rapid quenching leading to the formation of latent ion tracks [10,11]. In this work, we employed 120 MeV Ag ions for irradiation. Consequently, in addition to point defects, we anticipate the formation of ion tracks in thin films, which will influence their magnetic and electronic properties. Ion fluence values of 5 × 10^11^ ions/cm^2^, 1 × 10^12^ ions/cm^2^, and 5 × 10^12^ ions/cm^2^ were selected to induce multiple ion impact phenomena. This paper presents a comprehensive investigation of the electronic structure and magnetic properties using advanced techniques such as X-ray photoelectron spectroscopy (XPS) and superconducting quantum interference device-vibrating sample magnetometer (SQUID-VSM) on well-characterized pristine and irradiated PLD thin films. The primary objective of this investigation is to elucidate the role of ion irradiation treatment in tuning the magnetic and electronic properties.

## 2. Experimental Methods

In the initial phase of this study, we synthesized Ba_0.7_Sr_0.3_Fe_x_Ti_(1−x)_O_3_ pellets with x = 0, 0.1, and 0.2 intended for thin film preparation via the pulsed laser deposition (PLD) technique. The solid solution with the requisite stoichiometry was obtained through a solid-state reaction methodology. The precursor materials, namely i.e., BaCO_3_, SrCO_3_, Fe_2_O_3_, and TiO_2_, were meticulously weighed in precise stoichiometric ratios. These materials were subjected to ball milling using zirconia balls in a propanol medium for six hours at a rotational speed of 200 revolutions per minute (rpm). Following this, the ball-milled powder was calcined at 1000 °C for five hours to facilitate the formation of the perovskite phase. After calcination, the powder was combined with polyvinyl alcohol (PVA) and subjected to uniaxial hydraulic pressing at a pressure of 10 tons to form pellets. The PVA-integrated calcined powders were then pressed into targets with a diameter of 1 inch. To promote grain growth and densification, these hydraulically pressed samples underwent sintering at 1250 °C for 24 h. Utilizing these pellets as targets, Fe-doped BST films were deposited onto (100) oriented platinized silicon wafers through the PLD method. The stoichiometric rotating target was ablated using a short-pulsed KrF excimer laser with a wavelength of 248 nm (LAMBDA PHYSIK, Compex 201 model, Fort Lauderdale, FL, USA), operating at an energy of 220 mJ and a frequency of 10 Hz. Thin films were deposited under an oxygen pressure of 200 mTorr, with the substrate temperature maintained at 550 °C. The thickness of the films was consistently set at 100 nm. Subsequent characterization of the samples was conducted to evaluate their structural, morphological, and magnetic properties. Room temperature X-ray diffraction (XRD) patterns were acquired using Cu k_α_ radiation (λ = 1.54 Å, Bruker D8 advance, Billerica, MA, USA). The surface topography of the thin films was analyzed via atomic force microscopy (AFM, Parker, Cleveland, OH, USA). Magnetic measurements of the thin film samples were performed using a SQUID-VSM (T-Tesla MPMS SQUID—Vibrating Sample Magnetometer, Quantum Design, San Diego, CA, USA), with a normalized sample volume of approximately (10 × 10 × 0.5) mm^3^. Surface chemical states were investigated utilizing an ESCA-5000 (Physical Electronics, Chanhassen, MN, USA) Versa Probe X-ray photoelectron spectroscopy (XPS) system, employing an Al K_α_ (1486.7 eV) beam coupled with a 124 mm hemispherical electron analyzer. Calibration during XPS measurements was performed using the C 1s peak. The irradiation experiments were performed with 15 UD tandem pelletron accelerators at IUAC, India. The irradiation was performed with 120 MeV silver ions at three distinct fluences: 5 × 10^11^ ions/cm^2^, 1 × 10^12^ ions/cm^2^, and 5 × 10^12^ ions/cm^2^.

## 3. Results and Discussions

### 3.1. Structural Analysis

**Figure 1:**
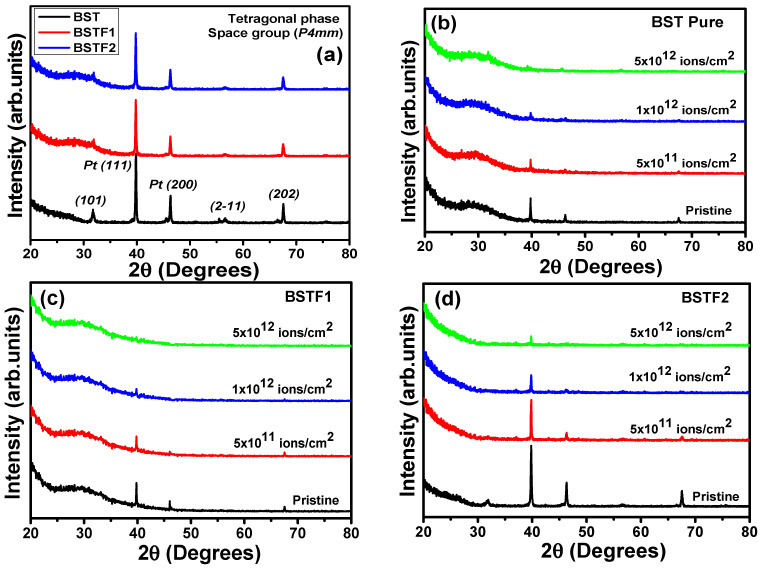
(**a**) XRD patterns for pristine BST, BSTF1, and BSTF2 thin films; (**b**–**d**) XRD patterns for 120 MeV Ag ion irradiated thin films with three different fluences (5 × 10^11^, 1 × 10^12^, 5 × 10^12^ ions/cm^2^).

In Figure 1a–d, the X-ray diffraction patterns for pristine and Ag ion irradiated BST, BSTF1, and BSTF2 thin films are shown. The Bragg’s reflection peaks are indexed in Figure 1a for pristine thin films. The indexing indicates that the pristine thin films have a tetragonal (space group *P4mm*) structure. The high-intensity (111) and (220) reflections are correlated with the platonized Si wafer substrate. The other reflections, i.e., (101), (2-11), and (202), correspond to the tetragonal perovskite phase. With Fe cationic doping, no significant change is observed in the XRD patterns. Figure 1b–d shows the XRD patterns for 120 MeV Ag ion-irradiated BST, BSTF1, and BSTF2 thin films, along with the pristine ones. The peak intensity of the reflections reduces with increasing ion fluence. For all three thin films, the (101), (2-11), and (202) reflections completely vanished for 5 × 10^12^ ions/cm^2^ fluence. Ion irradiation-induced modifications in the structural properties are correlated with the energy deposition mechanism of the 120 MeV Ag ions. As the energy range is high, the electronic energy loss (*S_e_*) is higher than the nuclear energy loss (*S_n_*). The calculated *S_e_* and *S_n_* values for 120 MeV Ag ion in tetragonal BaTiO_3_ from SRIM 2013 software are 2.11 keV/Å and 0.009 keV/Å, respectively. Here, the higher *S_e_* caused a significant change in the structural properties. The basic mechanism of such energy deposition to the host lattice via *S_e_* is explained with a standard thermal spike model [10,11]. The *S_e_* results in damage to the lattice by creating an ion track along with its trajectory and further causes a reduction in the peak intensity in XRD patterns. No 2θ shift is observed with Fe doping as well as with irradiation, which indicates the absence of any compressional or tensile shift in the lattice.

Upon penetration into a solid, energetic ions lose energy through two mechanisms: direct energy transfer to target nuclei via elastic collisions (nuclear energy loss (*S_n_*)) and ionization of the target atoms through inelastic collisions (electronic energy loss (*S_e_*)). In the present investigation, we focus primarily on *S_e_*, given the high energy of the irradiation ions (several MeV), while S_n_ becomes more significant in the keV range [12]. As the peak intensities (2-11) and (202) peaks of the tetragonal BST phase have reduced significantly, this provides a direct indication that multiple ion impact processes at high fluence have caused a generation of latent ion tracks due to the high *S_e_* of 120 Ag ions. For 5 × 10^12^ ions/cm^2^ fluence, (2-11) and (202) reflections have completely disappeared, which indicates amorphization. Apart from the perovskite phase, the peak intensity for Pt(111) and Pt(200) has reduced significantly with increasing irradiation fluence. This gives direct evidence that Ag ions have reached the substrate and damaged the lattice.

### 3.2. Microscopic Modification Observed from AFM Micrographs

**Figure 2:**
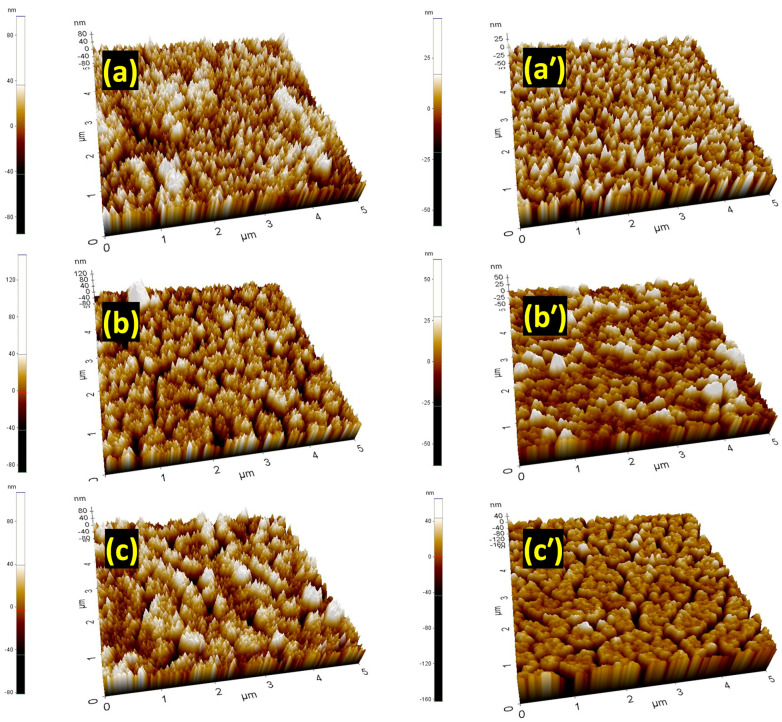
(**a**–**c**) AFM images for pristine BST, BSTF1, and BSTF2 thin films; (**a’**–**c’**) AFM images for 120 MeV Ag ion irradiated thin films with fluence 5 × 10^12^ ions/cm^2^.

In Figure 2a–c, the topographical surface features with atomic force microscopy (AFM) images are shown in a 5 μm scale for the pristine BST, BSTF1, and BSTF2 thin films. The same for Ag ion irradiated thin films with 5 × 10^12^ ions/cm^2^ fluence are shown in Figure 2a’–c’. With Fe doping, no significant change is observed in the topographical features, as all pristine thin films show a homogenized granular structure with almost similar height distribution. After irradiation, the average height of the grains was reduced. The height scale present on the left-hand side of all images makes it quite clear. The deposition of high *S_e_* might have caused such notable changes in the topography. Irradiation has not only reduced the average height of the grains, but also the distribution of grains has become more homogenous. Such a change in topographical features has been reported by Shukla et al. for PLD BiMn_2_O_5_ thin films [9]. Here, we cannot rule out the possibility of the generation of clusters of vacancies caused by *S_n_* [13]. In Figure 2c’, the observed porosity can be formed by a cylindrical damaged zone due to high *S_e_* [13]. The presence of such a porous topographical feature can be correlated with the reduced peak intensity of the reflections observed in XRD patterns.

### 3.3. Influence of Irradiation on Surface Oxidation States

**Figure 3:**
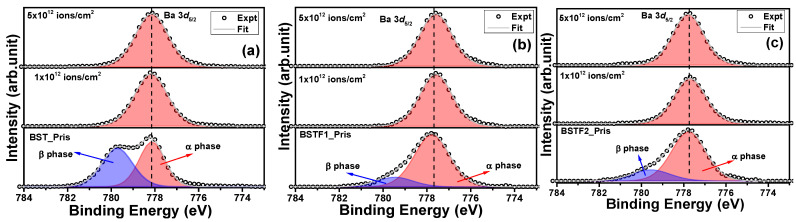
(**a**–**c**): Ba 3*d*_5/2_ peak for pristine and Ag ion irradiated BST, BSTF1, and BSTF2 thin films with 1 × 10^12^ and 5 × 10^12^ ions/cm^2^ fluence. α, and β phases correspond to the perovskite phase and barium carbonate/surface contaminations.

**Figure 4:**
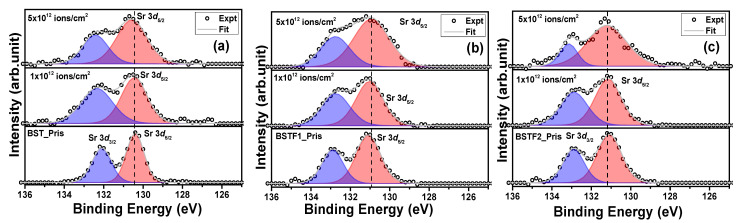
(**a**–**c**): Sr 3*d*_5/2_ and 3*d*_3/2_ peaks for pristine and Ag ion irradiated BST, BSTF1, and BSTF2 thin films with 1 × 10^12^ and 5 × 10^12^ ions/cm^2^ fluence.

Figure 3a–c shows the Ba 3d XPS peak of the BST, BSTF1, and BSTF2 thin films before and after Ag ion irradiation with 1 × 10^12^ ions/cm^2^ and 5 × 10^12^ ions/cm^2^ fluence. The accurate determination of chemical shifts due to irradiation necessitates precise identification of the peak position on the binding energy (BE) scale. To ensure consistency across our measurements, we calibrated all data using the C 1*s* peak at 284.6 eV. Peak fitting was performed using a mixed Lorentzian (30%) and Gaussian (70%) approach and Shirley’s background correction with the help of the CASA XPS software [14]. The analysis of the Ba 3*d*_5/2_ peak is shown in Figure 3a–c. For the pristine BST, BSTF1, and BSTF2 thin films, the Ba 3*d*_5/2_ peak was fitted with two distinct components corresponding to the α and β phases, located approximately at 778 eV and 780 eV, respectively [15]. Notably, the position of these components on the binding energy scale exhibits minor variations with increasing iron (Fe) doping concentrations, attributable to variations in the chemical environment. Our initial focus is to investigate the changes induced by electronic excitation. Remarkably, the β phase is absent in the irradiated samples of BST, BSTF1, and BSTF2 thin films. The higher BE component, identified as the α phase, is indicative of Ba atoms within the perovskite structure, whereas the β phase is associated with barium carbonate or surface contaminants [16]. Here, Ag ion irradiation has worked virtually as an etching agent, which removes the surface contamination layer. Apart from this, we observed minimal changes in peak position on the BE scale with increasing ion fluence. Figure 4a–c depicts the Sr 3*d* peak for all the thin films, including those subjected to ion irradiation. Each peak of the Sr 3*d*_5/2_ (130.4 eV) and 3*d*_3/2_ (132.1 eV) doublet is fitted with one component; no significant change in the peak position on the BE scale was observed, revealing no change in oxidation state due to Ag ion irradiation. Collectively, the observations from the Ba 3*d* and Sr 3*d* peaks suggest that Ag ion irradiation exerts minimal influence on the perovskite phase, which is in good agreement with the XRD observation.

**Figure 5:**
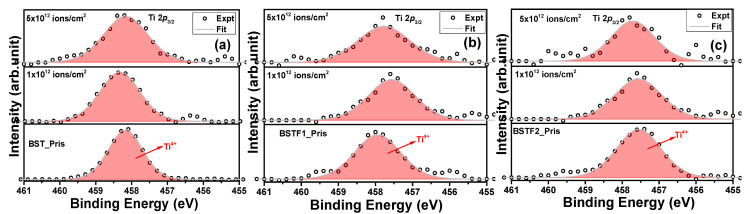
(**a**–**c**): Ti 2*p*_3/2_ peak for pristine and Ag ion irradiated BST, BSTF1, and BSTF2 thin films with 1 × 10^12^, and 5 × 10^12^ ions/cm^2^ fluence.

**Figure 6:**
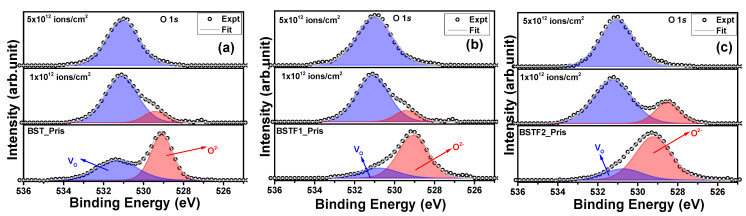
(**a**–**c**): O 1*s* peak for pristine and Ag ion irradiated BST, BSTF1, and BSTF2 thin films with 1 × 10^12^ and 5 × 10^12^ ions/cm^2^ fluence.

Figure 5a–c illustrates the Ti 2*p*_3/2_ peak for both pristine and Ag ion-irradiated thin films. The Ti 2*p*_3/2_ peak located at 458.2 eV corresponds to the Ti^4+^ charge state [15]. No changes in oxidation state were detected post-irradiation; however, the reduction in peak intensity may result from the rupture of Ti–O bonds induced by Ag ion irradiation. Similar to previous observations, no shifts in peak positions on the BE scale were recorded with increasing ion fluence. Figure 6a–c presents the O 1*s* peak for all the pristine and irradiated thin films. The peak fitting revealed two components corresponding to oxygen atoms and oxygen vacancies (V_O_) at 529 eV and 531.4 eV, respectively [15]. The presence of intrinsic V_O_ defects during the synthesis is evident [17]. In pristine thin films, both the perovskite phase and V_O_ components are observable, with the perovskite phase peak area exceeding that of the V_O_ peak. However, upon irradiation at a fluence of 1 × 10^12^ ions/cm^2^, the V_O_ peak area surpasses that of the perovskite phase, indicating significant rupture of Ti–O, Ba–O, Sr–O, and Fe–O bonds at the surface, thereby reducing the perovskite peak intensity and enhancing the V_O_ peak area. This observation highlights the generation of V_O_ as a direct consequence of Ag ion irradiation. At the highest fluence, 5 × 10^12^ ions/cm^2^, V_O_ predominance is observed across all three thin films, suggesting extensive rupture of the aforementioned bonds. Despite these observations, we avoid drawing a definitive conclusion regarding the potential compromise of all oxygen bonding within the perovskite phase. The impact of irradiation appears to be more pronounced on O atoms relative to other chemical elements of the perovskite structure. The absence of Bragg reflections corresponding to the perovskite phase in the XRD patterns at 5 × 10^12^ ions/cm^2^ fluence further supports the rupture of Ti–O, Ba–O, Sr–O, and Fe–O bonds, as well as the observations from O 1*s* photoemission. The increasing intensity of the oxygen vacancy component indicates that the rupture of Ti–O, Ba–O, Sr–O, and Fe–O bonds, as observed via X-ray photoelectron spectroscopy (XPS), is consistent with the reduction in intensity of the (2-11) and (202) peaks of the tetragonal BST phase in the XRD patterns. Both observations are indicative of latent track formation within the lattice, ultimately leading to the amorphization of the crystalline tetragonal BST phase (space group *P4mm*).

### 3.4. Irradiation-Induced Changes in Magnetism

**Figure 7:**
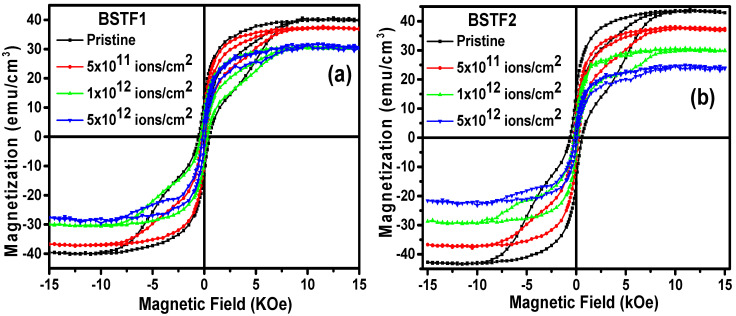
(**a**,**b**): Magnetization vs. magnetic field hysteresis loops for pristine and irradiated BSTF1 (**a**) and BSTF2 (**b**) thin films. The irradiation is performed with 120 MeV Ag ions with three different fluences (5 × 10^11^, 1 × 10^12^, and 5 × 10^12^ ions/cm^2^).

M vs. H curves are shown in Figure 7a,b for BSTF1 and BSTF2 pristine as well as irradiated thin films at RT. Ferromagnetic behavior is evident from the M vs. H hysteresis loop patterns. This weak ferromagnetism cannot be attributed to the presence of Fe_2_O_3_ or Fe_3_O_4_ phases. In the presence of Fe_2_O_3_ or Fe_3_O_4_ phases, the saturation magnetization (*M_s_*) value would have increased beyond 70 emu/cm^3^ [18]. We observe that there is no significant difference between *M_s_* for BSTF1 and BSTF2 thin films. The *M_s_* values for BSTF1 and BSTF2 thin films are 39.7 and 43.1 emu/cm^3^. The XRD patterns also do not indicate any presence of Fe_2_O_3_ or Fe_3_O_4_ phases as impurity phases. Therefore, the possibility of Fe_2_O_3_ or Fe_3_O_4_ phases-induced ferromagnetism can be ruled out. Fe doping-induced ferromagnetism is anticipated to be intrinsic. It is reported that with Fe doping, the cationic substitution of Ti with Fe results in the generation of V_O_ [18]. These V_O_ can trap electrons and act as an electron trap center. It has been reported by Coey et al. for diluted magnetic semiconductors (DMS) that such trapped electrons spend most of their time around V_O_ [19]. Those trapped electrons in the occupied orbital overlap with the *d* shell of the Fe magnetic dopant ion and create an impurity band. Such an overlap leads to the generation of bound magnetic polarons (BMP) and results in a long-range ferromagnetic ordering. Therefore, the formation of BMP causes such intrinsic long-range ferromagnetic ordering [19,20]. The Ag ion irradiation has deteriorated the magnetism value and does not show any radical change in the magnetic phase of the materials. The *M_s_* and magnetic coercivity values were reduced systematically with increasing ion irradiation fluence. The magnetic coercivity values for pristine BSTF1 and with 5 × 10^11^ ions/cm^2^, 1 × 10^12^ ions/cm^2^, and 5 × 10^12^ ions/cm^2^ irradiation doses were 0.54 kilo-Oersted (kOe), 0.35 kOe, 0.21 kOe, and 0.15 kOe, respectively. For BSTF2 thin films, the corresponding values were 0.61 kOe, 0.24 kOe, 0.22 kOe, and 0.15 kOe, respectively. In summary, the magnetic properties deteriorate with increasing irradiation fluence, indicating that irradiation disrupts the magnetic structure. Qualitatively, it can be said that the defect engineering-induced modifications in the nearest-neighbor interaction are responsible for this decline in magnetism. It is likely that the formation of latent tracks and associated defects has compromised BMPs, further degrading long-range ferromagnetic ordering with increased ion fluence. Consequently, electronic excitation neither completely disrupts existing magnetic ordering nor facilitates the emergence of new magnetic phases. If such transformations were to occur, one would expect radical changes and a complete reconstruction of the material’s phase, potentially transitioning from crystalline-to-crystalline or crystalline-to-amorphous states. Prior studies, such as those by Qin et al., have indicated that irradiation-induced defects, including oxygen vacancies, can induce ferromagnetism [21]. Our XPS observations corroborate the presence of VO; however, the high energy and multiple ion impacts suggest that cation interstitial defects may overshadow the effects of VO in influencing ferromagnetism, leading to an overall degradation in magnetic properties. High-energy irradiation can result in the formation of latent tracks or phase transitions, contingent upon the threshold electronic energy loss (S_eth_) [22,23]. The formation of latent tracks has been described through two primary models: the Coulomb explosion model, which is predicated on electrostatic repulsive forces [24,25], and the thermal spike model, wherein energy is imparted to lattice atoms, causing melting and subsequent quenching to form tracks [26,27]. The latter model is more widely supported in the literature. Upon penetration into a solid, energetic ions dissipate energy through two mechanisms: direct energy transfer to target nuclei via elastic collisions (nuclear energy loss, *S_n_*) and ionization of target atoms through inelastic collisions (electronic energy loss, *S_e_*). The present investigation emphasizes Se, given the high energy of the irradiation ions (several MeV), while Sn is more relevant in the keV range [12]. The significant reduction in the peak intensities of the (2-11) and (202) reflections of the tetragonal BST phase in XRD patterns provides direct evidence that multiple ions’ impact at high fluence resulted in the generation of latent ion tracks due to Se. This formation of latent ion tracks leads to amorphization within the lattice. Furthermore, the XRD patterns reveal a marked reduction in the intensity of the Pt(111) and Pt(200) substrate reflections with increasing fluence, indicating that latent tracks due to high electronic energy loss are formed not only in the thin film region but also within the substrate. The generation of cation interstitial defects in the amorphized lattice is expected, particularly as temperatures surpass the melting threshold in the vicinity of ion tracks. This leads to the formation of point defects near latent tracks, resulting in a compromised lattice structure. To maintain overall charge neutrality, the electrostatic Coulomb attraction fosters the formation of defect complexes such as ${Ti}^{4+}-V_{o}^{∎∎}$ [28]. In the context of electronic structure, O 2p orbitals dominate the valence band maxima (VBM), while Ti 3d orbitals dominate the conduction band minima (CBM) [21]. The irradiation process induces defects such as VO, which have low formation energies, allowing electrons from the VBM to be excited to the CBM, where they can be captured by Ti^4+^ cations. The formation of defect complexes, such as ${Ti}^{4+}-V_{o}^{∎∎}$, may play a crucial role in overshadowing the effects of di-positive V_O_ further contributing to the observed degradation in ferromagnetism [21]. The presence of the Ti^4+^ oxidation state was confirmed by XPS, supporting the possibility of defect complex formation. However, the lack of irradiation-based molecular dynamics simulations limits our ability to quantitatively assess the formation energy of such defect complexes, as these calculations are resource-intensive. Consequently, the concept of defect complex formation in barium strontium titanate (BST) thin films has been introduced based on the existing literature, recognizing that quantitative assessments regarding the formation of these complexes exceed the scope of this manuscript. Additionally, it is pertinent to note that cross-sectional transmission electron microscopy characterization could yield significant insights within the framework of this investigation. Such high-resolution imaging techniques are capable of revealing information about latent tracks and interstitial point defects. However, the primary aim of this study is to explore the modulation of magnetic properties through electronic excitation, underpinned by structural and electronic property analyses. In scenarios where enhanced magnetism can be achieved through optimized defect engineering, high-resolution imaging will be instrumental in elucidating the underlying microscopic dynamics. This experimental investigation demonstrates that electronic excitation serves as a viable approach for tailoring magnetic properties in strongly correlated systems.

## 4. Conclusions

Doping and 120 MeV Ag ion irradiation techniques have been used as tools for controlled defect engineering to tailor the ferromagnetism in pulsed laser-deposited Ba_0.7_Sr_0.3_Fe_x_Ti_(1−x)_O_3_ thin films with x = 0, 0.1, and 0.2. At higher ion irradiation fluences, significant lattice damage is evident from the XRD patterns. The high electronic energy loss (*S_e_*) damages the lattice by generating ion tracks along the ion trajectory, resulting in a reduction in peak intensity in the XRD patterns. The diminished intensity of reflections from platinized silicon wafer substrates confirms that latent track formation due to high *S_e_* has occurred in the substrate as well. Atomic force microscopy (AFM) images reveal that the average grain height has decreased, and the size distribution has become more homogeneous with irradiation. The presence of porous regions among grains corroborates the reduction in the intensity of the reflections observed in the XRD patterns. Although XPS indicates an increasing concentration of V_O_, ferromagnetism consistently diminishes with escalating irradiation fluence. The formation of latent tracks due to high *S_e_* and multiple ion impacts is suggested as the primary factor disrupting BMPs and contributing to the deterioration of magnetic properties. This study demonstrates that the application of 120 MeV Ag ion irradiation at three different fluences does not optimize controlled defect engineering for the enhancement of magnetism. Further exploration of shallow-level defects, such as oxygen vacancies, or achieving a complete crystalline-to-crystalline phase transformation with distinct dynamics may improve magnetic properties. Future sophisticated experiments are warranted to optimize the choice of irradiating ion species, ion energy, and fluence to enhance magnetism. The insights regarding changes in electronic structure and their correlation with magnetic properties due to electronic excitation elucidated in this work are significant for anticipating modifications resulting from high *S_e_* deposition in other systems.

## Data Availability

The original contributions presented in this study are included in the article. Further inquiries can be directed to the corresponding author.
